# Combination Regimen With Lithium and Antipsychotic in Bipolar Manic Episodes: Impact on Adult Hospitalization Length of Stay

**DOI:** 10.7759/cureus.8568

**Published:** 2020-06-11

**Authors:** Hyun Kyung Lee, Shruti Prabhudesai, Ramu Vadukapuram, Noha Eskander, Rikinkumar S Patel

**Affiliations:** 1 Psychiatry, Hanyang University College of Medicine, Seoul, KOR; 2 Psychiatry, Rajarshi Chhatrapati Shahu Maharaj Government Medical College, Kolhapur, IND; 3 Psychiatry, State University of New York Upstate Medical University, Syracuse, USA; 4 Psychiatry, California Institute of Behavioral Neurosciences & Psychology, Fairfield, USA; 5 Psychiatry, Griffin Memorial Hospital, Norman, USA

**Keywords:** bipolar disorders, manic episodes, lithium, antipsychotics, inpatient psychiatry, length of stay, mood disorders

## Abstract

Objective

To discern the demographic predictors in bipolar disorder (BD) manic patients receiving combination regimen, that is, lithium and antipsychotic, and to study the impact of a combination regimen on hospitalization length of stay (LOS) and total charges.

Methods

We used the nationwide inpatient sample (NIS) and included 1,435 adult inpatients with BD, manic episodes, and receiving lithium. Independent sample T-test with equality measures was used for LOS and total charges. Logistic regression model was used to find the odds ratio (OR) for the combination regimen to estimate the predictors with 95% CI.

Results

Among the inpatient sample, 34.5% received a combination regimen. There was statistically no significant difference between the combination regimen versus non-combination regimen cohorts by age and sex. A higher proportion of inpatients receiving combination regimen were from high-income families above 75th percentile (56.4%) and covered by private insurance (47.5%). Blacks (OR 2.00, 95% CI 1.43-2.82) and hispanic (OR 2.31, 95% CI 1.49-3.57) had higher odds of receiving a combination regimen compared to whites. The combination regimen significantly reduced LOS for BD, manic episode management by 2.8 days (95% CI 1.13-4.53 days, P < 0.001). There was statistically no significant mean difference in total charges (P = 0.495).

Conclusion

A combination regimen with lithium and antipsychotics significantly reduced LOS for BD manic episodes by 2.8 days compared to inpatients receiving lithium monotherapy. So, starting the combination regimen from the initial day of hospitalization should be considered as an effective model for faster response.

## Introduction

Bipolar disorder (BD) is a chronic debilitating psychiatric illness involving episodes of mania and/or hypomania and depression [1]. The prevalence of BD in the United States (US) is 2.8% and similar in both adult males (2.9%) and females (2.8%) [2]. Current treatment options for BD manic episodes include mood stabilizer (lithium, valproate or carbamazepine), second-generation antipsychotics (SGAs), and benzodiazepines [3]. Lithium is the gold-standard treatment with its acute anti-manic efficacy in combination with antipsychotics, and for long term maintenance treatment through inhibition of intracellular signaling proteins and modulation of dopamine, glutamine and gamma-aminobutyric acid (GABA) neurotransmission [4,5].

According to the 2018 guidelines from the Canadian Network for Mood and Anxiety Treatments (CANMAT) and the International Society for Bipolar Disorders (ISBD), the first-line pharmacological treatment for BD manic episodes can be decided between monotherapy and combination therapy based on the necessity of rapid response, severity of mania, previous history of response, and tolerability concerns. First-line treatment includes monotherapy with lithium, quetiapine, valproate, asenapine, or aripiprazole (recommended to try first in the order), or combination therapy with SGAs (quetiapine, risperidone, aripiprazole, or asenapine) and lithium or valproate [6]. The combination therapy has a better response (about 20%) than monotherapy with mood stabilizer, and is preferred when the faster response is needed, in severe manic episodes, or in history of partial response to monotherapy, unless there are tolerability concerns [7,8]. Treatment-resistant BD manic episodes are treated with electroconvulsive therapy (ECT) and should not be considered as a "last resort" in the treatment algorithm as earlier use of ECT reduces hospitalization stay and total cost [9].

Combination treatment of SGAs with lithium has been provided greater efficacy than lithium monotherapy for manic and mixed BD episodes, and reduces acute illness morbidity. The addition of SGAs during the first week of treatment significantly improves manic symptoms, and also prevents re-hospitalization during the one-year period after a BD manic episode. SGAs are efficacious for long-term maintenance therapy for BD mania, and reduces relapse rate due to potent efficacy, and better compliance [10,11]. Due to the antagonistic action of SGAs on serotonin 2A (5-HT2A) receptor and dopamine D2 receptor leads to increased dopamine in the mesolimbic system that improves manic symptoms [12].

Our goal is to conduct a cross-sectional inpatient study to discern the demographic predictors for starting a combination regimen in patients with BD manic episodes by adding antipsychotic medication with lithium. Next, our goal is to study the impact of the combination regimen on hospitalization length of stay (LOS) and total charges.

## Materials and methods

We used the nationwide inpatient sample (NIS), the largest de-identified data from the US hospitals, and does not require institutional review board approval [13]. We included 1,435 adult inpatients (age 18 years and above) with a primary diagnosis of BD manic episode using the International Classification of Diseases, ninth revision (ICD-9) diagnosis codes: 296.00-296.06, 296.10-296.16, 296.40-296.46, 296.60-296.66 or 296.81, and receiving lithium (ICD-9 procedure code, 94.22). They were further grouped based on receiving antipsychotics (ICD-9 procedure code, 94.23), that is, lithium plus antipsychotic as a combination regimen.

Demographic variables included were age (18-35, 36-50 and 51-65 years), sex (male and female), race (white, black, hispanic and others), median household income (as per national percentiles), and primary insurance (medicare, medicaid, private and self-pay/uninsured) [14]. We also included the LOS and total charges (in US dollars, $) during hospitalization for BD, manic episode, and LOS was calculated as the total number of inpatient nights [14].

We used descriptive statistics and Pearson’s chi-square test to determine differences in subgroups by patients’ demographic. Independent sample T-test and T-test for equality measures were used for continuous data, that is, LOS and total charges. The logistic regression model was used to find the odds ratio (OR) for the combination regimen to estimate the predictors with 95% CI. We used the Statistical Package for the Social Sciences (SPSS) version 26 (IBM Corporation, Armonk, NY) in the study with statistical significance set at P ≤ 0.05.

## Results

Our study inpatients were primarily young adults (18-35 years, 55.4%) and whites (54.1%) from high-income families above 75th percentile (37.5%). Out of 1,435 inpatients, 34.5% (N = 495) additionally received antipsychotic with lithium, that is, combination regimen.

There was statistically no significant difference between those receiving combination regimen versus non-combination regime cohorts by age and sex distribution. Whites (56.6%), blacks (22.2%), and hispanics (14.1%) were managed by the combination regimen. A higher proportion of inpatients receiving combination regimen were from high-income families above 75th percentile (56.4%) and covered by private insurances (47.5%) as shown in Table 1.

**Table 1:** Demographic distribution of study inpatients by receiving combination regimen

Variable	Combination regimen		Total	P-value
	(-)	(+)		
Total N	940	495	1435	-
Age at admission, %				
18 – 35 years	55.3	55.6	55.4	0.099
36 – 50 years	25.5	29.3	26.8	
51 – 65 years	19.2	15.1	17.8	
Sex, %				
Male	48.9	50.5	49.5	0.572
Female	51.1	49.5	50.5	
Race, %				
White	52.7	56.6	54.1	<0.001
Black	22.0	22.2	22.1	
Hispanic	10.4	14.1	11.7	
Others	14.9	7.1	12.1	
Median household income in percentiles, %				
0 – 25^th^	30.2	11.7	23.4	<0.001
26^th^ – 50^th^	19.8	17.0	18.8	
51^st^ – 75^th^	23.5	14.9	20.3	
76^th^ – 100^th^	26.5	56.4	37.5	
Insurance, %				
Medicare	21.9	22.2	22.0	<0.001
Medicaid	39.0	14.1	30.4	
Private	26.7	47.5	33.9	
Self-pay/uninsured	12.3	16.2	13.6	

Combination regimen was utilized majorly in whites, yet in regression model blacks (OR 2.00, 95% CI 1.43-2.82) and hispanic (OR 2.31, 95% CI 1.49-3.57) had two times higher odds for being managed with combination regimen compared to whites. Inpatients from high-income families above the 75th percentile had five times higher odds (95% CI 3.28-7.43) for the combination regimen. Insurance status was a statistically non-significant predictor for the combination regimen as shown in Table 2.

**Table 2:** Predictors of combination regimen in study inpatients

Variable	Odds ratio	95% CI		P-value
		Lower	Upper	
Race				
White	Reference			
Black	2.00	1.43	2.82	<0.001
Hispanic	2.31	1.49	3.57	<0.001
Others	0.61	0.39	0.96	0.032
Median household income in percentiles				
0 – 25^th^	Reference			
26^th^ – 50^th^	2.53	1.64	3.89	<0.001
51^st^ – 75^th^	1.67	1.08	2.58	0.022
76^th^ – 100^th^	4.94	3.28	7.43	<0.001
Insurance				
Medicare	Reference			
Medicaid	0.39	0.26	0.58	<0.001
Private	1.38	0.96	1.98	0.081
Self-pay/uninsured	1.04	0.67	1.62	0.860

The combination regimen significantly reduced the LOS for BD manic episode management by 2.8 days (95% CI 1.13-4.53), whereas there was statistically no significant difference in mean difference in total charges as shown in Table 3.

**Table 3:** Impact of combination regimen on hospitalization stay and total charges $: United States dollars

Variable	Combination regimen	
	(-)	(+)
Length of stay, in days		
Mean ± SD	14.3 ± 18.25	11.5 ± 8.57
Mean difference	2.83	
95% CI	1.13 to 4.53	
P-value	0.001	
Total charges, in $		
Mean ± SD	38555 ± 81877	41560 ± 29213
Mean difference	-3004	
95% CI	-11629 to 5261	
P-value	0.495	

## Discussion

We found that blacks and hispanics have a two times higher likelihood of receiving a combination regimen with lithium and antipsychotic compared to whites. The utilization of the combination regimen was associated with a statistically significant reduction in LOS by 2.8 days compared to those who only receive lithium.

Cookson reported that the antipsychotics are widely prescribed in 72% to 92% of bipolar manic patients in outpatient settings [15]. We found a lower rate of utilization of antipsychotics in the inpatient population (34.5%) compared to the outpatient study. Despite females having a higher risk of psychiatric comorbidities, we did not see a statistically significant difference in the utilization of a combination regimen based on gender [16,17]. A combination regimen was majorly utilized in whites (56.6%) followed by blacks (22.2%) and hispanics (14.1%). Considering that the CANMAT and ISBD 2018 guidelines for BD management, combination regimen usage decision is based on the severity of mania [6]. So, the racial disparity of manic severity could affect the difference in the utilization rate of the combination regimen across the races [18,19]. However, a community-based study by Perron et al. reported that the expression and functional impairments of BD are very similar across racial/ethnic groups [20]. A higher proportion of whites received a combination regimen probably could be due to racial disparity in receiving BD treatment [21]. Yet, in the adjusted regression model, we found that the blacks and hispanics had two times higher odds of receiving a combination regimen compared to whites. This pattern is supported by previous studies including Depp et al. which states that black patients and hispanics were more likely than non-Latino Whites to receive pharmacotherapy with antipsychotics for BD management [22].

BD is the most expensive diagnosis in behavioral health, both for patients with BD and the insurance companies [23]. Patients with BD utilize about three- to four-fold more healthcare resources and incur more than four-fold greater healthcare costs than patients without BD [24]. Our study shows that the patients covered by private insurances had a 38% higher likelihood of being managed with a combination regimen than those by public insurances. Also, patients from high-income families had five times higher likelihood of receiving a combination regimen than those from low-income families. There is insufficient data for supporting the impact of insurance or socioeconomic status (SES) on adding antipsychotics to mood stabilizers until now. Considering that the choice of medication can also be influenced by cost restrictions and combination regimen is more expensive than monotherapy, the availability of insurance has a possibility to affect starting a combination regimen on the patient [25,26].

The use of the combination regimen significantly reduced the LOS of BD patients by about 2.8 days per inpatient hospitalization, but statistically no significant difference in total charges. Hospitalization stay may be reduced due to higher efficacy and faster onset of action of the combination regimen compared to monotherapy [11]. Clinical trials suggest that about 20% more patients will respond to a combination regimen, and studies also have shown that the combination regimen reduces relapse rate and re-hospitalization for BD [7]. A meta‐analysis found that combination regimen with haloperidol, olanzapine, risperidone, and quetiapine was significantly more effective than monotherapy with a mood stabilizer for managing BD manic episodes, but combination regimen was less well-tolerated than monotherapy [27]. The recurrence time of events (mania, depression, or mixed) after a manic episode was longer with those managed with combination regimen, especially with atypical antipsychotics in combination with lithium/valproate, than with placebo. The combination regimen exhibited more side effects than monotherapy, and safety and tolerability patterns differ between types of combination like tremor, weight gain (with olanzapine), increased sedation (with quetiapine, haloperidol, and asenapine) and akathisia (with aripiprazole) [7].

This study has some limitations due to the use of the administrative NIS data as it does not have information on patient-related variables. The data does not include information about other concomitant medications administered to the patients and does not provide information on the type of antipsychotics used for managing BD. BD manic episodes were identified using the ICD-9 diagnostic codes and not rating scales. Also, the study sample was sub-grouped based on antipsychotics that were identified by ICD-9 procedure code, which may lead to underreporting of antipsychotic use. However, our study provides information to the field with data on why adding antipsychotics to lithium can be an effective model, and its impact on LOS and charges during hospitalization. Also, it represents an inpatient sample, that is, the clinical population as practiced across the United States, which can influence clinical decision-making.

## Conclusions

The combination regimen significantly reduced LOS for BD manic episodes by 2.8 days compared to inpatients receiving lithium monotherapy. So, starting the combination regimen by adding antipsychotics to lithium or any other mood stabilizers from the initial day of hospitalization should be considered as an effective model for faster response.
